# 基于分子网络技术识别新污染物及其转化产物的研究进展

**DOI:** 10.3724/SP.J.1123.2024.03014

**Published:** 2025-01-08

**Authors:** Xiaomei TAN, Yuwei ZHANG, Zhaoyu JIAO, Nanyang YU, Si WEI

**Affiliations:** 南京大学环境学院,污染控制与资源化研究国家重点实验室, 江苏 南京 210046; State Key Laboratory of Pollution Control & Resource Reuse, School of Environment, Nanjing University, Nanjing 210046, China

**Keywords:** 高分辨率串联质谱, 分子网络, 新污染物, 转化产物, high-resolution tandem mass spectrometry, molecular networking, emerging contaminants, transformation products

## Abstract

环境中新污染物及其转化产物分布广泛且风险未知。其种类繁多且结构复杂,使得传统的靶向分析和可疑物筛查分析难以满足分析需求。高分辨质谱技术的发展促进了基于液相色谱-高分辨率串联质谱的非靶向分析在环境领域的应用,尤其在识别未知转化产物方面有所贡献。然而,非靶向分析面临着如何从复杂数据中找到关注化合物及其转化产物的挑战。分子网络反映质谱之间的相似性,将结构彼此相似的分子家族成簇,实现海量质谱数据集的可视化和社区化,并通过网络和社区对污染物进行传播注释,可以全局展示和系统理解复杂串联质谱数据集,为非靶向分析提供了新方向。本文介绍了基于高分辨率串联质谱的分子网络分析方法及其在新污染物的非靶向筛查中的应用,包括技术原理、工作流程、应用现状和未来展望。本文重点展示了分子网络技术在药物、全氟化合物和消毒副产物等新污染物及其转化产物中的应用状况,强调分子网络技术普遍适用于各种环境介质中新污染物的筛查、揭示环境中污染物的全貌、推进化合物的环境行为和毒理学特性的研究。

## Abstract (en)

Emerging contaminants and their transformation products are widely distributed in the environment. These pollutants carry unknown risks owing to their persistence, migration, and toxicity. The wide variety and complex structures of these substances render them difficult to identify using only target analysis. Suspect screening analysis can identify more substances than target analysis in a single run. However, this analysis method is based on limited data and cannot meet the growing demand for compound identification, especially for emerging contaminants and their transformation products with unknown information. The development of high-resolution mass spectrometry technology has promoted the applications of nontarget analysis in the environmental field, especially for identifying unknown transformation products. At present, the challenges of nontarget analysis include the difficulty of finding compounds of interest and their transformation products from complex data. Molecular networking calculates the similarity between mass spectra based on an improved cosine similarity algorithm. This method can cluster molecular families with similar structures, achieve visualization and a collection of massive mass spectral datasets, and promote the annotation of pollutants through networks and communities. Molecular networking can globally organize and systematically interpret complex tandem mass spectral datasets, providing a new direction for nontarget analysis. This technology was first used in proteomics and gradually introduced into metabolomics for the discovery of new natural products. Recently, it has been introduced into the environmental field for the study of various man-made chemicals, particularly for the discovery of emerging contaminants and their transformation products. In this paper, we introduce a molecular networking analysis method based on high-resolution tandem mass spectrometry and describe its applications in the nontargeted screening of emerging contaminants, focusing on the technical principles, workflow, application status, and future development prospects. This paper discusses the applications of molecular networking technology in the detection of emerging contaminants and their transformation products such as drugs, perfluorinated compounds, and disinfection byproducts. Molecular networking technology is widely applicable to the screening of emerging contaminants in various environmental media, revealing the full range of pollutants in the environment and promoting studies on the environmental behavior and toxicological properties of these compounds.

新污染物是指对生态环境或者人体健康存在较大风险,但尚未纳入环境管理的物质。常见的新污染物包括抗生素、内分泌干扰物以及全氟/多氟烷基化合物(per- and polyfluoroalkyl substances, PFASs)在内的持久性有机污染物,这些物质在多种环境介质中被检出^[1⇓-3]^,对环境和人体健康造成巨大的风险^[4,5]^。这些物质在环境中可能发生进一步转化,生成未知的转化产物^[6]^。研究表明,部分抗生素转化产物的毒性比母体化合物更强^[7,8]^,某些转化产物在环境中还会反向转化成母体化合物^[9]^,而非进一步被降解。因此,识别新污染物的转化产物已经成为重要的环境问题之一。

新污染物及其转化产物种类繁多、结构复杂,传统的靶向分析方法由于通量低且依赖于标准品,难以满足新污染物的分析需求。随着高分辨质谱的发展,非靶向分析逐渐成为新污染物分析的重要手段。高分辨率串联质谱能够提供精确质量数、同位素模式、碎片质量数等基本信息,帮助确定分子式和进一步阐明化学结构。高分辨率质谱提供的庞大信息能够帮助研究人员一次性鉴定母体和转化产物,而不需要重复实验^[10]^。液相色谱-高分辨率串联质谱(LC-HRMS/MS)作为有机污染物检测的常用仪器,推动了非靶向筛查技术在新污染物识别中的应用,尤其在识别未知转化产物方面。然而非靶向分析获取的质谱信息复杂,数据量大,如何从大量的质谱信息中找到关注的化合物及其转化产物,是当前非靶向分析面临的重要挑战^[11]^。

Bandeira等^[12]^提出了分子网络的概念,基于质谱间的谱图相似性对数据聚类,为非靶向分析提供了新方向。分子网络是一种强大的工具,可提供对复杂LC-MS/MS数据集的全局展示和系统理解^[13]^,其最初主要用于生命科学、生物分析和药物研究中的小分子结构鉴定,后来被引入环境非靶向分析体系,用于识别环境中未知的化合物及其转化产物^[14]^,揭示复杂环境污染背后的化学空间^[15]^。本文对基于高分辨率串联质谱的分子网络分析方法及其在新污染物和转化产物中的应用进行了介绍。首先总结了分子网络的技术原理和研究进展,然后围绕新污染物及其转化产物的分析,概述了工作流程,并综述了分子网络技术在新污染物及其转化产物识别中的应用,最后对分子网络在环境新污染物及其转化产物研究中的未来发展进行了展望。

## 1 基于高分辨率串联质谱的分子网络分析方法

串联质谱技术的发展提高了MS/MS谱图的采集速率^[16]^,然而基于有限的质谱数据库进行数据库比对检索的方法^[17]^已不能满足人们快速鉴定大量物质的需求。2007年,Bandeira等^[18]^提出“谱图对(spectral pairs)”的概念,谱图对可以实现在没有数据库的基础上进行物质的检索和鉴定,开辟了基于质谱数据鉴定未知物质的新方向。此概念最初用于蛋白质的鉴定工作中,将鉴定沿谱图传播,从而检测到意外修饰和多重修饰的肽。谱图对进一步组合形成“质谱网络”^[12]^,即分子网络的雏形。相较于传统的数据库检索,质谱网络试图将相关物质的不同谱图相关联,这填补了传统方法中丢失的物质间的重要关联信息,为依赖数据库进行蛋白质鉴定打开了新的思路,允许有关新物质的发现。

Watrous等^[19]^将质谱分子网络技术引入代谢组学中,监测微生物群落中的小分子变化,深入了解细菌发育过程。改进的余弦相似性算法被引入谱图相似性打分体系^[19]^,分子网络将相似的谱图聚集,从而实现更高效的数据挖掘和化合物鉴定,同时可以辅助监测目标化合物的修饰和降解。质谱分子网络技术为蛋白质组学和代谢组学中的物质分析开辟了新的计算方法。2013年,分子网络的概念正式被Pieter等^[20]^总结提出,之后Pieter等^[21]^构建了开源使用分子网络的GNPS(Global Natural Products Social Molecular Networking)平台,使得分子网络技术能够被广泛应用于基于质谱的代谢组学研究。由于庞大的质谱数据的共享和开源使用,GNPS也成为目前主流的分子网络分析平台。分子网络通过质谱比对而构建,以找到具有相似碎裂模式的谱图。除了解析肽分子,该技术还可鉴定非肽分子,包括具有非线性结构的天然产物、脂质、聚糖和其他化合物家族^[22]^。应用于微生物代谢产物研究的分子网络根据具有不同作用方式的基因亚网络,发现了4个非核糖体肽合成酶衍生的分子家族^[23]^。该研究发现了此前未报道的切除、糖基化、水解和生物合成反应的中间体及其副产物,有助于更深入地研究微生物的生物合成能力^[24]^。为了研究环境中的抗菌转化产物及其潜在的健康风险,Yu等^[25]^把分子网络应用于制药废水进行非靶向筛选和鉴定,发现了30个未在环境中报道的抗菌转化产物,涉及羟基化、脱氨、烷基化、氧化和乙酰化等反应。这些研究表明,分子网络分析的范围已经扩展到环境领域。母体化合物及其转化产物可以看作具有某类结构特征的分子家族,因此,将分子网络技术应用于转化产物的研究十分具有前景。

### 1.1 分子网络技术的原理

数据驱动的分子网络技术的原理主要基于结构和谱图紧密关联的假设。假设分子在串联质谱分析中获得的碎裂谱图与其原始化学结构相关^[26]^,具有相似结构的分子将表现出相似的串联质谱图。因此,复杂混合物中所有谱图之间的谱图相似性可以外推到混合物中分子之间的结构相似性。通过计算得到的谱图相似性矩阵可以进一步可视化为分子网络,其中每个节点都是一张质谱图,节点之间的边表示高于用户定义的相似性评分阈值的谱图相似性。传统的谱图相似性算法为点积算法,即通过余弦分数将每个串联质谱图简化为多维空间中的一个矢量,其中每个维度对应于碎片离子的质荷比(*m/z*)及其离子强度,然后计算空间中两个谱图矢量之间的角度,以表示两个谱图之间的相似性^[27]^。

作为分子网络分析的主要平台,GNPS采用修正的余弦相似性算法计算谱图相似性。修正的余弦相似性算法不仅考虑了碎片的原始*m/z*,同时考虑到中性损失值,即前体离子与碎片离子的差值。其背后的基本原理在于对结构的单一修饰通常会导致谱图中的片段峰子集随修饰的*m/z*位移而移动(例如,-18表示失去一个水分子)。此原理的引入促使了串联质谱图分析思路的转变。网络分析方法开始考虑谱图之间的关系,不仅可以实现与参考谱图的匹配,还能够将实验谱图对齐后通过相似性匹配成对。通过谱图相似性对谱图进行分组,分子网络可以识别与网络理论中的社区(或簇)相对应的分子家族^[26]^。分子家族是结构彼此相似的分子簇,为进一步的数据分析提供了多种优势,可实现海量质谱数据集的可视化和社区化,以及通过网络和社区对污染物进行传播注释^[20]^。

### 1.2 分子网络技术的发展

GNPS平台最初构建的分子网络工作流是基于MS-Cluster算法的“经典分子网络(Classical MN)”。MS-Cluster算法可以依赖上传数据中的前体质量和二级谱图信息将样本数据中同一物质的谱图进行快速聚类,并在聚类中选择具有最高信噪比的代表性谱图或得到具有高信噪比的虚拟谱图作为“共识谱图(a consensus spectrum)”,从而大大缩减成网规模,降低数据分析难度^[28]^。该种聚类策略仅依赖有限质谱信息完成数据过滤,极易造成同分异构体识别的缺失。

基于此,GNPS平台在2020年引入了基于特征的分子网络(FBMN)^[29]^作为辅助工具以解决以上问题。基于特征的分子网络不通过MS-Cluster算法进行批量聚类,而是利用成熟的质谱处理软件(如MS-DIAL^[30]^、MZmine^[31]^、XCMS^[32]^等)的功能,在对平台上传数据前完成对一级质谱信息(如同位素模式和保留时间)和离子淌度信息的整合工作,进而从更多信息维度对物质进行分离,改进经典分子网络。借助外部工具处理所得的质谱信息,FBMN增加了以下功能:(1)通过色谱或离子淌度信息分离产生类似二级谱图的异构体;(2)谱图注释;(3)纳入相对定量信息,从而实现稳健的下游代谢组学统计分析。Robin等将FBMN用于分析4个具有不同氧化还原条件的河岸过滤点的水样,对地表水和饮用水中的有机微污染物及其转化产物进行可靠的鉴定,注释了43种具有不同降解速率的抗高血压药物,包含4种新的转化产物^[14]^。然而,物质在质谱电离的真实环境中可以产生具有不同碎裂模式的多个离子种类(如质子化和钠化等),这导致构建的分子网络中同一化合物的不同加合型式分子无法聚簇,即产生不必要的分子家族分离,限制了基于网络的数据库传播注释。

为了克服以上瓶颈,Schmid等^[33]^在FBMN的基础上开发了离子特征分子网络(IIMN),将色谱峰形相关分析整合到分子网络中,以连接同一分子的不同种类离子,以达到去冗余的目的。

以上3种类型的分子网络受限于谱图相似性算法,无法得到分子家族间的关联信息。Olivon等^[34]^开发的通过整合两种不同算法来生成分子网络的软件MetGem则可以克服基于余弦的质谱相似性分数的不足。MetGem允许通过两种算法对质谱数据集进行分析,一种是基于GNPS平台的经典分子网络(MN)算法,另一种基于随机邻居嵌入(t-SNE)算法^[35]^。t-SNE算法是一种用于高维数据可视化的技术,通过捕捉高维空间中的局部相似性进行降维,同时尽量保留全局结构。基于密切相关的集群之间的距离,研究者可探讨集群间的关系。

国内外研究还评估了不同采集参数对基于分子网络的非靶向筛查技术应用于海洋、河流和土壤样品分析的影响^[36]^。Paolo等^[37]^比较了网络节点总数、多变量聚类以及与频谱质量相关的指标,发现Q Exactive平台的自动增益控制、显微扫描、质量分辨能力和动态排除是最关键的参数,有助于分子网络在不同场景中应用参数的选择。

分子网络作为非靶向分析中的一环,在进行物质筛查之后,还需要借助其他工具鉴定和评估物质以达到转化产物等新污染物的全方位风险评估和危害分析。分子网络之后的工作重点落在结构鉴定之上。分子网络帮助我们找到更多关注的转化产物,谱图相似性部分反映了结构相似性,也帮助我们进行物质鉴定。但是结构鉴定是一项巨大的挑战,需要依靠计算机工具的进步来降低鉴定工作的难度,提高鉴定准确度,加快物质鉴定速度。

目前,GNPS平台托管了用于注释和其他相关工作流程的大型谱库,可以直接从分子网络启动^[17]^。此外,已经开发了计算机模拟质谱裂解的方法来克服现有数据库中可用质谱数据较少所带来的局限性。Allard等^[38]^通过使用CFM-ID^[39]^将天然产物数据库(DNP)中的海量化合物在计算机中模拟质谱碎片化,建立了计算机数据库(ISDB),使得谱图数据库规模激增到原来的10倍以上,极大程度地拓展了可用的谱图数据。同理,该方法可以迁移到环境物质模拟谱图的生成中,以构建污染物及其转化产物的计算机数据库。Gordon等^[40]^报道了PFASs非靶向筛查的新方法,该方法通过精心策划的结构列表和计算机预测生物/非生物转化产物来构建PFASs化学结构数据库,结合计算机生成PFASs的串联质谱数据库和分子网络,对环境样品中的PFASs进行全面筛查和高通量注释。随后,Ricardo等^[41]^开发的基于分子网络的自动化扩展注释工具NAP(Network Annotation Propagation)被GNPS平台引入,该工具将计算机碎片算法MetFrag^[42]^与网络拓扑结构相结合,计算从化合物结构数据库中检索的候选结构与邻近节点的相似性,对候选结构重新排名,在一定程度上提高了计算机预测候选列表中正确结构的可能性。

此外,分子网络节点使用来自多种工具的多类信息进行增强^[17]^,与物质的先验知识结合,通过集成多维度信息,扩大分子网络的应用领域。除了数据驱动的分子网络策略,Zhu等开发的知识驱动的多层网络技术(KGMN)^[43]^,具备从已知物鉴定未知物的能力,并显著提升了物质鉴定的准确度。该技术利用代谢反应网络中产物和底物存在结构相似性和二级谱图相似性的基本原理,设计了代谢物二级谱图“谱图借用”和“多次迭代”算法,利用标准质谱数据库鉴定出的已知物作为种子节点,依靠代谢反应网络进行注释的迭代和传递,突破了标准二级谱图数据的覆盖度限制,利用较小的数据集就可实现大规模的已知代谢物鉴定,给分子网络的构建提供了一种新思路。全局网络优化方法(NetID)^[44]^也基于加合物质量差、碎片、同位素以及可靠的生化转换关系连接质谱峰,并进行全局网络优化,通过线性规划策略产生最优且一致的网络注释结果。

分子网络基于谱图相似性连接未知物质和已知物质,是一种非常流行、用途广泛且富有洞察力的谱图组织方法,其参与的非靶向分析发展迅猛。未来的发展重点是通过集成多维度的数据和知识信息^[45]^实现知识从已知物到未知物的传播,例如对节点添加前体质量位移、化学类别和其他参考数据信息,以及添加节点间的差异信息,便于推测未知峰对应的物质结构和来源^[46]^。在分子网络提供的局部谱图连接与低维嵌入的全局相似性结构视图之间建立联系,有望大大提高非靶向分析的便利性,具有广阔的研究前景^[17]^。

### 1.3 分子网络鉴定转化产物的常规分析流程

基于分子网络分析转化产物常规的分析步骤主要包含质谱数据获取、分子网络构建和物质鉴定三部分。

具体流程如下。首先采取适当的方法制备样品,主要是分离、提取、浓缩等步骤。其次是质谱数据的获取和转换,常规环境样品的数据通常使用液相色谱-高分辨率串联质谱获取;不同仪器采集的格式不同,可通过MSconvert软件^[47]^将不一致的质谱数据转换成需要的数据格式。接着进行数据预处理,使用开源软件(MS-DIAL、MZmine、XCMS、OPENMS^[48]^等)对质谱特征进行检测和提取。经过预处理的数据输入到平台(GNPS、MetGem等)计算谱图相似性,构建分子网络并利用可视化软件(Cytoscape^[49]^等)呈现分子网络。通常,转化产物研究采用分子网络技术构建分子家族后,与本地数据库或者开源的标准质谱数据库匹配,可以从复杂混合物的质谱网络中注释节点,实现物质注释的自动化^[50]^,此类节点被称为已知节点。除此之外,分子网络还可以通过分子家族将已知某类结构作为基本骨架或者特征离子,通过网络传播注释相邻节点,从而预测未知转化产物的结构,指导下一步的研究。从数据预处理开始后的每一个步骤都可以通过实验数据库和计算机注释工具(SIRIUS^[51]^、MS2LDA^[52]^、MetWork^[53]^、NAP等)对分子网络中的各个节点进行注释。通过各类别的数据库检索检验注释结果,可发现不曾出现在数据库中的未知转化产物或者新污染物。然后基于评估策略和先进的智能决策工具对复杂组分中鉴定到的化合物的重要性进行排序和评估,以此找到重点关注的转化产物。最后使用标准品进行验证。

## 2 分子网络技术识别新污染物及其转化产物的研究进展

新污染物及其转化产物在环境中的发生和分布已经成为一个全球性问题,并且对人类健康和环境造成了潜在危害。当前,分子网络作为一种相对成熟的技术,通过大规模的污染物质谱数据库及网络传播注释,将极大程度地提升污染物注释效率,挖掘发现此前无法鉴定的未知物质。已有研究将分子网络技术应用到环境领域,显著提升了从环境样品尤其是水样中发现新污染物的效率。目前基于分子网络的分析方法已经被应用于药物、PFASs、饮用水消毒副产物(DBPs)、毒素等新污染物及其转化产物的研究中。这些研究表明,分子网络技术能够在没有先验信息的情况下仅依赖串联质谱数据将结构相似的物质聚成分子家族,从已知节点识别未知节点,发现新物质,尤其是难以被识别的低丰度化合物。然而,串联质谱谱图的噪声影响成网。针对这些问题进行研究可以改善分子网络的结果。随着技术的逐步完善,分子网络在环境转化产物研究中的应用会被持续推进。

### 2.1 分子网络在识别药物及其转化产物中的应用

分子网络技术起步于蛋白质组学,在代谢组学研究中逐渐成熟,在天然产物的鉴定分析中广泛应用^[50]^。最开始的研究聚焦在具有潜在药物作用的新型生物活性化合物上^[54]^。作为细菌和真菌的次生代谢物^[55]^,抗生素、抗肿瘤药物和降胆固醇药物等在分子网络的助力下被不断发现^[56]^。然而,新型药物被发现可能具有持久性、迁移性和毒性(PMT)^[57]^,新型药物的使用及其未知转化行为为环境带来了新的风险,其中抗生素及其转化产物由于其可能的危害已被列为重点关注的新污染物,对药物及其转化产物的分析鉴定十分重要。

Yu等^[25]^开发了一种基于分子网络的非靶向筛选策略,运用该策略从污水处理厂排放的废水中检出了52种抗菌药物和49种转化产物,其中有30种新型抗生素转化产物尚未在环境相关研究中报道。该研究发现的转化反应类型包括羟基化,脱氨基、烷基化、二氯化、去甲基化、氧化和乙酰化。并且基于REACH法规对抗生素及其转化产物进行PMT评估,该研究发现大多数转化产物的PMT等级均高于母体化合物,表明新型抗生素转化产物具有不可忽视的潜在风险。孙卫玲等^[58]^根据分子网络应用于抗生素及其转化产物的研究申请了名为“同时包含已知和潜在未知转化途径的抗生素转化产物识别方法”的专利。

Wu等^[59]^选取5种结构相似的磺胺类药物作为靶向新污染物,通过基于分子网络的非靶向筛选方法探索其在实验室规模废水生物处理实验中的转化模式,最终鉴定出45种转化产物,其中包括新发现的14种转化产物。在此基础上,该研究通过转化途径比较、反应频率分析和显性转化产物比较,总结了磺胺类药物的5种具体转化模式。结果表明,废水中的磺胺类药物普遍发生蝶呤螯合和甲酰化(主要转化途径)以及乙酰化、甲基化和脱氨反应,其中,甲酰化作为废水中磺胺类药物转化的主要转化途径被首次报道。基于同样的非靶向筛查模式,该团队利用分子网络检测了全球高消耗的抗抑郁药西酞普兰和舍曲林在废水中的转化产物^[60]^,包括9个新发现的转化产物。结果表明,与以往非靶向策略比较,分子网络策略在候选转化产物的优先排序和新转化产物挖掘方面表现更出色,尤其针对低丰度转化产物。后续此方法还揭示了抗抑郁药氟西汀和文拉法辛在废水中的优势转化产物和途径^[61]^。

Qian等^[62]^使用网络框架将非靶向分析和配对质量距离(paired mass distance, PMD)结合,开发了简化网络分析(simplified network analysis, SNA)方法,以揭示废水中复杂的转化过程。SNA可以提取废水中常见的分子结构和相应的转化途径。研究通过SNA揭示了中国15个污水处理厂废水中不同结构的新污染物的转化途径,总共鉴定出4种抗高血压药物及22种转化产物。研究显示废水的各个处理环节中去除难易程度不同的污染物之间存在明显的结构差异。研究还发现,出水中80.81%的产物的质谱峰面积高于母体,强调了转化产物风险评估的重要性。此研究表明根据结构选择性地采用废水处理技术有助于消除废水处理厂中顽固的新污染物。

国外的研究团队也利用基于特征的分子网络对地表水和饮用水中的有机微污染物及其转化产物进行高置信度的鉴定。Robin等^[14]^首先基于分子网络对饮用水相关和季节依赖性化合物进行了优先级排序和分子间连接;随后在得到的分子网络中,对43种化合物进行了注释,其中包括卡马西平和沙坦类药物的4种新的转化产物,并对沙坦及其转化产物在不同氧化还原条件和季节下的去除行为进行了分组。

这一系列的研究表明,分子网络在水样中阐明药物转化产物的优势突出,能够辅助构建和完善环境中药物的监管治理体系。

### 2.2 分子网络在识别PFASs及其转化产物中的应用

PFASs是一类合成化学品,可用作表面活性剂、抗染色剂和防粘剂等,产量颇高。PFASs的毒性和环境持久性是全球关注的问题。为了解决PFASs禁用的问题,工业界开发了许多视为商业机密的替代PFASs。下一代替代品的结构及其环境危害往往是未知的。2020年发表在*Science*期刊上的文章通过基于质谱的非靶向检测方法研究土壤中不同化学结构的氯代全氟聚醚羧酸盐及其地理分布,推动了对新型PFASs的进一步关注^[63]^。已有的PFASs筛选工作流程受到相对较小的分子数据库和普遍缺乏的谱图数据库的限制,未能广泛应用。

基于cfmR平台的新方法^[40]^展示了分子网络技术和计算机预测谱图数据库在PFASs及其转化产物研究中的实用性。该方法使用基于机器学习的竞争性碎裂建模(CFM)算法^[64]^,预测所有PFASs的串联质谱碎裂谱图,并使用内部化合物集合训练了一个新的CFM预测模型。进一步通过MSnbase^[65]^计算实验和预测质谱之间的谱图相似性来构建分子网络,连接潜在的PFASs母体-产物对,揭示复杂环境介质中未被发现或者新出现的PFASs。水解作为大多数PFASs的主要非生物转化过程,在该研究中得到充分探究。结果表明,近一半的母体-产物对的反应遵循热力学或动力学反应规则。不遵循常规的母体-产物对能够辅助识别非典型反应条件下产生的新型PFASs。

因此,当在环境样品中鉴定出以前未知的PFASs时,分子网络可以通过已知PFASs与合理的转化途径的共现增加未知物结构注释的证据权重,帮助PFASs识别、连接和环境归宿研究。

### 2.3 分子网络在识别微藻毒素及其转化产物中的应用

近年来,海洋中藻类暴发^[66]^,其中包括许多产毒微藻。这些藻类以产生亲脂性和热稳定性的毒素而闻名。这些毒素可以通过海洋食物链转移并积累在贝类、螃蟹和其他水产品中^[67]^,最终导致食用这些产品的消费者发生急性疾病事件,同时这些毒素还可能对人体健康造成长期影响,引起癌症的发生。因此,科学家们一直关注水产品中藻毒素的组成及其构成的潜在威胁。

早在2015年,国外的研究人员采用分子网络的方法对西雅图地区夏季藻华爆发阶段的蓝藻毒素进行了研究,发现了3种新的环肽类毒素变体,约占水华中毒素含量的1/2^[68]^。利用分子网络对中国黄海北部和南海的5种利玛原甲藻菌株的细胞内毒素谱进行了非靶向分析^[69]^,共鉴定出恐龙毒素(DTX1)的24个脂类转化物和1个立体异构体,其中15个酯类似物被首次报道。分子网络助力毒素的全面识别,并指明所有菌株均表现出特异性的毒素谱,以及毒素谱可能与区域相关。此外,小鼠急性毒性实验表明脂化毒素毒性高于游离毒素。因此,毒素转化产物更应该得到紧迫关注。

### 2.4 分子网络在识别DBPs中的应用

饮用水消毒过程中的一个重大问题是天然有机物发生氯化反应,形成多种消毒副产物^[70]^,其中大多数DBPs含有至少一个氯原子,称为氯化消毒副产物(Cl-DBPs)。流行病学研究表明,长期暴露于DBPs与不良健康影响(包括膀胱癌、发育影响和流产)之间存在潜在关联^[71]^。然而,现有的知识未能明晰导致毒性的特定Cl-DBPs。由于化学物质的多样性、复杂的背景信号以及高分辨质谱仪器固有的分析波动,传统的同位素模式、质量缺陷和直接分子式预测不足以准确识别真实环境样品中的各种Cl-DBPs。研究表明,未鉴定的Cl-DBPs可能对观察到的毒性有重大贡献,这突显了对新物质结构鉴定的迫切需要,促进水样中Cl-DBPs的识别和分析^[72]^。

Huan等^[73]^提出了一种结合机器学习与分子网络的识别含氯化学物质的新策略。分层机器学习框架包含两个基于随机森林的模型,模型使用来自PubChem^[74]^的140余万个分子式训练,在对NIST20的14000多个唯一分子式的评估中,达到93.3%的含氯分子式识别准确率和92.9%的精准含氯数量的准确率。训练好的模型被集成到ChloroDBPFinder中。ChloroDBPFinder注释识别已知和未知的含氯化合物之后,结合分子网络对筛查物质进行结构注释。在与自来水中阿斯巴甜氯化相关的现有Cl-DBPs数据集上进行测试的结果显示,ChloroDBPFinder有效地提取了159个特征化合物,并通过分子网络初步注释了10个Cl-DBPs的结构。此研究团队还研究了多种串联质谱数据处理策略,这些策略均能与分子网络结合提升物质注释效率。团队探讨了嵌合串联质谱谱图对谱图相似性分析和分子网络的影响,提出了DNMS2Purifier^[75]^的谱图清洗流程,以便自动化提取代谢特征、分配串联质谱谱图并清洗谱图。该研究是对现有基于色谱图谱和质谱数据库方法的补充,并有效改善了谱图匹配率和分子网络结果。

可见,分子网络推动串联质谱数据注释的同时,也促进了质谱数据预处理的发展,在相互促进中形成更完整的体系,而不断优化的分子网络应当在环境转化产物的研究中得到更大发展空间。

### 2.5 分子网络在识别其他新污染物及其转化产物中的应用

除了关注单一类别污染物及其转化产物,分子网络还被用于水样中多种污染物的检测。Pradeep等^[76]^结合分子网络技术对表面活性剂、塑料添加剂、橡胶和树脂等化合物的同一类别的物质进行聚类,在澳大利亚的一条河流中检出15种污染物。许衎等^[77]^为全面筛查长江下游江苏段的潜在新污染物,耦合质谱数据库和分子网络,共识别出118种新污染物,涉及农药、抗生素、阻燃剂和表面活性剂等多种类别。该筛查策略能够在无先验信息和标准品的情况下高通量鉴定污染物,加快水环境中新型与未知污染物的系统化发现。Daniel等^[78]^利用FBMN开发了液相色谱耦合质谱的先进工作流程,允许对来自大规模空间调查的海水物质组成进行可扩展的比较和映射,以及未知化合物的分子家族注释。研究表明,有机物化学型在极端降雨后发生了显著变化,这种转变的分子驱动因素可归因于多种合成化学品,强调了人为影响。此数据分析流程为未来海洋生态系统的分子制图和监测提供了一个可扩展的框架,将有助于对化学污染如何影响海洋进行评估。

此外,分子网络也拓展到不同环境介质中或者不同场景下进行污染物监测,包括人组织样品、动物、植物、微生物、土壤、大气等环境样品的研究,这表明分子网络对环境样品具有普适性。Brendan等使用分子网络筛选出两种喹硫平在人体内的代谢产物^[79]^。Allegra等^[80]^将分子网络用于挖掘从患者肺部分离的喹诺酮类药物的转化产物,在肺组织中发现了4种转化产物。Hu等^[81]^结合点击化学-质谱探针(CC-MS)和分子网络技术深入分析了大鼠体内邻苯二甲酸二(2-乙基己基)酯(DEHP)的代谢足迹。该研究筛选出247个潜在的DEHP代谢物,通过分子网络注释了4种代谢物类型(邻苯二甲酸单乙基己基酯衍生物、2-乙基己醇衍生物、葡糖苷酸偶联物和邻苯二甲酸酯二酯类物质),并提出一种可能的大鼠DEHP代谢的互补途径。Wang等^[82]^构建了化学衍生化-分子网络方法,揭示肠道微生物群-宿主共代谢中的羧化代谢组,包括脂肪酸、胆汁酸、*N*-酰基氨基酸、苯并杂环酸、芳香酸和其他未知的小规模分子簇,表明分子网络可以关联环境小分子与代谢物质,促进环境与健康的关联研究。Hu等^[83]^通过基于傅里叶变换离子回旋共振质谱(FT-ICR-MS)的反应组学和分子网络分析,研究了污泥生物炭衍生的溶解有机物(DOMs)在污泥热解下的转化途径。研究发现母体-产物对的数量随着热解温度的降低而增加。网络分析揭示了转化过程中DOMs的复杂性,阐明了DOMs生成的潜在机制,为污泥热解工艺优化和有害物质控制提供了理论支持。Papazian等^[84]^通过集成开源化学信息学和计算工作流程,利用分子网络实现大气颗粒物中有机化合物的广泛分子表征,包括遗留的持久性有机污染物、多环芳烃(PAHs)、增塑剂、杀虫剂和相关的转化中间体,促进了分子水平上对污染空气来源和影响的理解。Alexander等^[85]^使用分子网络探索了室内环境气体中多个化学家族如何受到人类活动的影响,发现由于人类居住而积累的挥发物部分似乎比建筑环境本身排放的部分对人类健康更有害,为医院或办公室环境的改善提供参考。

这些研究表明,分子网络技术已经在多种环境转化产物的分析中被广泛应用,并且有良好的发展前景。

## 3 总结和展望

分子网络为环境转化产物以及新污染物的研究提供了新的思路。将分子网络技术应用到环境分析中,研究者能对环境中新污染物及其转化产物进行快速、高效、全面地识别和鉴定,完善环境风险评估并改进污水处理工艺。然而,分子网络最初用于天然产物的挖掘,而环境领域关注的物质大部分是人为产生的,两者并不完全一致。因此,目前应用于环境领域的分子网络技术仍然存在以下不足之处,在日后的发展中需要完善。(1)分子网络对于谱图相似性的表征还有待改进,以提高结构相似性物质聚类的准确度。(2)针对部分类别新污染物成网率不佳的问题,分子网络技术可以叠加该类污染物的类别特征来针对性促进成网。(3)在网络传播注释方面,结果的假阳性率仍然较高,同样可以结合单一类别污染物的其他信息来提高注释准确率。总之,未来的分子网络技术还需集成多维度的数据和知识信息,实现知识从已知物到未知物的传播,加快物质鉴定速度,提高鉴定准确度,以揭示环境中污染物的全貌,推进化合物的环境行为和毒理学特性的研究。
